# Turbulence drives arteriovenous remodeling: an experimentally validated multi-scale model of neointimal hyperplasia

**DOI:** 10.1088/1361-6560/ae1ac8

**Published:** 2025-11-20

**Authors:** M Alyssa Varsanik, Carly Thaxton, Duc Nguyen, Joseph A Pugar, Sanjeev Dhara, Willa Li, Nhung Nguyen, Alan Dardik, Luka Pocivavsek

**Affiliations:** 1 Section of Vascular Surgery, Department of Surgery, University of Chicago Medicine, Chicago, IL, United States of America; 2 Vascular Biology and Therapeutics Program, Yale School of Medicine, New Haven, CT, United States of America; 3 Department of Surgery, Yale School of Medicine, New Haven, CT, United States of America; 4 Division of Vascular Surgery, Department of Surgery, Emory University, DeKalb County, GA, United States of America; 5 Department of Surgery, The Icahn School of Medicine, Mount Sinai, NY, United States of America

**Keywords:** Arteriovenous fistula (AVF), neointimal hyperplasia, turbulence, hemodynamics, computational fluid dynamics, agent based model, aortocaval mouse model

## Abstract

**Objectives.:**

Arteriovenous fistula (AVF) failure is a frequent clinical problem among end stage renal patients seeking durable long term dialysis access. The most common histological *in vivo* observation of AVF failure is endothelial injury at the juxta-anastomosis area (JAA) followed by thrombus deposition and subsequent neointimal hyperplasia (NH). While hemodynamic factors have been postulated to affect AVF remodeling and failure, the spatial correlations between changes in hemodynamics post AVF creation and *in vivo* physiologic observations remain poorly understood. In this work, we developed a novel computational fluid dynamics (CFDs) model of an AVF using a pre-established aortocaval mouse model and integrated it with agent-based modeling (ABM) for NH.

**Approach.:**

The CFD simulation was performed using an animal-specific aortocaval fistula geometry derived from *in vivo* CTA images with prescribed boundary conditions (BCs) obtained from *in vivo* ultrasound measurements. CFD results were validated against *in vivo* ultrasound velocity measurements at the level of the fistula. CFD allowed quantification of turbulence intensities throughout the fluid domain of the AVF.

**Results.:**

Turbulence was significantly elevated at the JAA and in regions of venous outflow stenosis. Turbulence intensity served as an input parameter for a simple two-rule ABM to test the hypothesis that non-homeostatic hemodynamic changes resulting from AVF creation drive spatial gradients in endothelial damage and proliferation of vascular smooth muscle cells leading to an increase in venous thickness or NH.

**Significance.:**

Our findings show that increased velocity and turbulence in the JAA parallels *in vivo* NH formation, and that further from the JAA (both cranial and caudal) velocity and turbulence decrease incrementally. The results corroborate that perturbed hemodynamics in the JAA are potential triggers for NH and the source of thickness gradients observed in AVFs.

## 1. Introduction

Vascular access is a lifeline for patients who require chronic hemodialysis, but the durability of arteriovenous fistulas (AVFs) remains suboptimal (Kanterman et al 1995, Asif et al 2006, Duque et al 2017, Martinez et al 2018, Venkat Ramanan et al 2022, USRDS 2024) (Asif et al 2006, Duque et al 2017). The autologous AVF is the preferred and most common way that vascular surgeons establish dialysis access for patients with end stage renal disease (ESRD). While the prevalence of ESRD continues to rise, the life expectancy of ESRD patients also increases, with approximately 85% of ESRD patients requiring dialysis for continued care (USRDS 2024). The AVF serves as a vital access point for the efficient removal and return of blood during dialysis. While AVF is the preferred choice over alternative access methods given their superior patency rates, AVF creation is associated with early and late failures, and an overall failure rate of 60% (Venkat Ramanan et al 2022). The primary cause of fistula failure is occlusive venous stenosis downstream of the fistula connection (Kanterman et al 1995). These failures necessitate reintervention, and in many cases, the only remaining solution is to ligate and reposition the access site entirely. Despite the magnitude of the clinical challenge, there are no surgical techniques or medical therapies to effectively prevent AVF failure.

The AVF fistula is a unique system that bypasses the high-resistance vessels, ultimately providing high flow rates that facilitate efficient dialysis. The high arterial flow rates entering the venous system create highly complex hemodynamic forces acting within the vein. These velocity and pressure fluctuations are further augmented by the cardiac cycle: systole and diastole. When combined with the intricate geometries of the arterial and venous anastomosis, blood flow transitions from a laminar to turbulent state, a physical phenomenon often identified by the palpable sensation of a ‘thrill’ felt on the arm over the fistula. The complexity in the AVF fluid flow dynamics presents a challenge for analyzing AVF remodeling and failure.

The current lack of AVF improvement is largely due to incomplete understanding of AVF pathology and hemodynamics. As such, both experimental and computational modeling approaches have been conducted to explore the multifactorial and multi-scale nature of AVF failure. *In vivo* studies have shown an association between AVF failure and peri-anastomotic neointimal hyperplasia (NH) and medial fibrosis (Kanterman et al 1995, Rotmans et al 2003, Allon et al 2011, Simone et al 2014, Wong et al 2014, Hall et al 2015, Martinez et al 2018). Additionally, computational modeling of fistulas has played a crucial role in advancing our understanding of fistula hemodynamics and the associated challenges with fistula maturation and patency. Studies have shown that changes in wall shear stress (WSS), pressure, and pulsatility after AVF creation may promote areas of endothelial activation that lead to stenosis and thrombosis (Fan and Karino 2010, Chiu and Chien 2011, Ene-Iordache and Remuzzi 2012, Gimbrone and García-Cardeña 2013, Bozzetto et al 2016, Hammes et al 2021). Specifically, computational fluid dynamic (CFD) models reconstructed from intravascular ultrasound and 2D venograms of the cephalic vein arch in humans showed that the inner wall of the cephalic bend was characterized by low flow velocity and wall pressure, suggesting a possible hemodynamic explanation for stenosis and recurrent thrombosis (Hammes et al 2021). In another study of AVFs created in canines, CFD analysis showed that NH is associated with low and disturbed WSS (Chiu and Chien 2011). There is growing evidence that AVF failure is instigated by hemodynamic changes specifically pulsatile laminar WSS and disturbed flow which perturb the endothelium and may induce selective expression of atherogenic and thrombotic genes, ultimately triggering the formation of NH (Fan and Karino 2010, Chiu and Chien 2011, Ene-Iordache and Remuzzi 2012, Gimbrone and García-Cardeña 2013, Bozzetto et al 2016). Other studies have used human ultrasound measurements to define their CFD models in exploring the turbulence as a function of radiocephalic fistula angle (Abbott et al 2021). The clinically ideal (desired) mechanobiological response of an AVF is uniform dilation of the vein. However, focal stenosis driven by NH often leads to further perturbation of AVF flows and the need for secondary fistula interventions. The spatial distribution of NH in AVFs has long been correlated to hemodynamics. In particular, the cephalic arch and the juxta-anastomosis area (JAA) are highly prone to both hemodynamic perturbations and NH driven stenosis. Perturbed flow drives stenosis which drives further fluid perturbation generating a postulated vicious cycle of maladaptation. In summary, prior data underscore the critical role of hemodynamic factors in NH formation and AVF failure. Yet the direct correlation between vascular pathophysiology and fluid dynamics changes post AVF creation remains ambiguous and necessitates the need to quantify hemodynamics in parallel with neointimal changes *in vivo*. Further, the value of predictive modeling for AVF failure has yet to be defined.

To study hemodynamic effects on biological responses, modeling approaches such as Agent Base Models (ABMs) provide a powerful tool to explore the underlying cellular responses and activities within a global spatial geometry (Corti et al 2021). ABMs utilize a system biology approach to capture the intrinsic behavior through imposing basic, probabilistic rules tuned to mimic native cell behavior. The response of the cellular system naturally emerges from describing the dynamics of individual agents (e.g. cells’ mitosis, apoptosis …), agent-agent and agent-environment interactions (Zhang et al 2009, Soheilypour and Mofrad 2018, Montagud et al 2021). Using this bottom-up approach, many studies have successfully replicated vascular adaptation processes using ABMs including cellular proliferation, atherosclerosis, in-stent restenosis, and bypass graft remodeling (Corti et al 2020, 2021, 2022a, 2022b, Colombo et al 2021). Nevertheless, due to the incomplete understanding of hemodynamics changes in AVF, to date negligible efforts have focused on establishing a direct link between the complex behavior of the endothelium and the varying flow conditions in AVF through an integration of CFD and ABM.

The aim of the present study is to characterize blood flow in the geometry of an *in vivo* AVF model, with a focus on velocity, static pressure, and turbulent intensity profiles, specifically within the vein. Our goal is to quantify changes in these hemodynamic parameters in parallel with the endothelial changes that have been previously published in the mouse aortocaval fistula model (Yamamoto et al 2013b). Here we construct and validate a CFD model of an animal-specific aortocaval fistula and quantify hemodynamic changes on the venous side in the fistula model. Detailed analyses of the effects of fistula stenosis and venous stenosis on hemodynamics are presented to correlate changes in AVF configurations with the flow dynamics. Hemodynamic parameters are then used as inputs for ABM rules to model NH. The results of the ABM model are validated against experimental results.

## 2. Materials and methods

### 2.1. AVF model and animal surgical procedures

Animal experiments were conducted in full adherence to federal guidelines and with the authorization of the Institutional Animal Care and Use Committee at Yale University. Mouse experiments were conducted as described in previous studies (Yamamoto et al 2013a, 2013b, Bai et al 2024).

**Figure 1.**
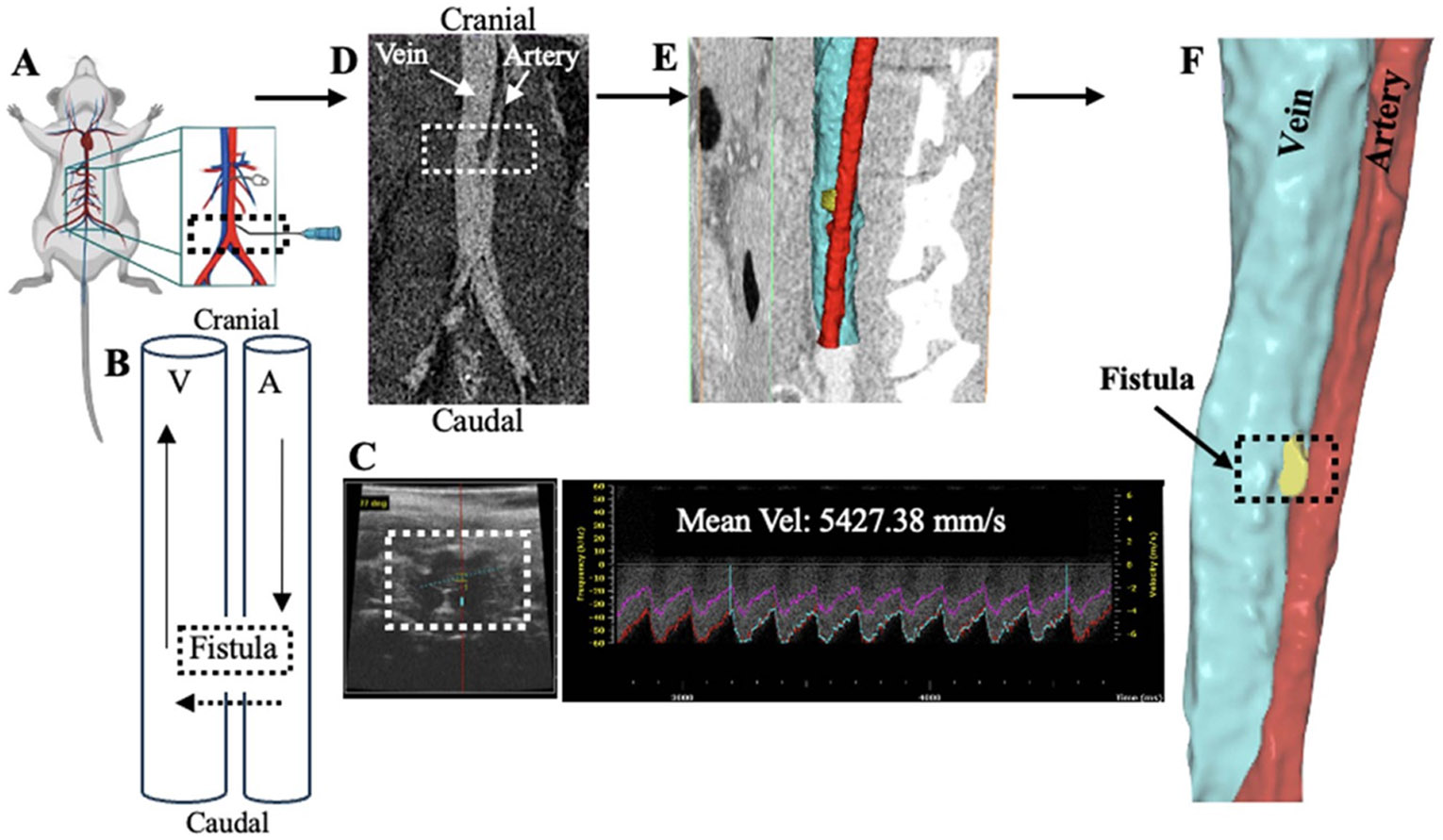
(A) Schematic representation of the aortocaval puncture fistula model. (B) Illustrative schematic of the native blood flow direction (solid arrows) in the aorta from cranial to caudal, in the IVC from caudal to cranial direction, and the blood flow across the fistula (dotted arrow) from artery (A) to vein (V) as a result of the aortocaval puncture. (C) Representative ultrasound assessment of the patent fistula (left) and mean velocity measurement of blood flow through the fistula (right). D) CTA DICOM image from *in vivo* aortocaval fistula with vein (left), artery (right) and fistula (dotted white box). (E) Three-dimensional segmentation of CTA DICOM aortocaval fistula preserves global geometry for the CFD simulations. (F) Final 3D segmentation, vein (blue), artery (red), fistula connection (yellow).

Briefly, the mouse abdominal cavity was entered via midline laparotomy incision from xiphoid to pubic symphysis. Abdominal contents were reflected to the right to expose the inferior vena cava (IVC) and aorta below the renal arteries. A 25-gauge needle was used to puncture the IVC through to the aorta, creating the AVF (figure 1(A)). The common technique of using adjacent tissue for compression to achieve hemostasis was adopted. Initial technical success of AVF creation was verified through direct observation of pulsatile flow. The abdomen was then closed. The AVF blood flow pattern is described in figure 1(B). To study the effects of central venous stenosis on AVF hemodynamics, data was sourced from a previously published experiment from the Dardik lab (Taniguchi et al 2020).

### 2.2. *In vivo* ultrasound measurements

Following the methods previously described in Bai et al (2024) for confirming AVF patency and measuring blood flow velocities, Doppler ultrasound (Vevo 770 and Vevo F2 High-Resolution Imaging System; Fujifilm Visual Sonics, Inc., Toronto, Canada) using probe RMV704 (40 MHz) was conducted at baseline and serially postoperatively (figure 1(C).)

### 2.3. *In vivo* CT imaging of the global AVF geometry

Adopting the methods previously described in Bai et al (2024), CT scan of the AVF post creation (specifically, postoperative day 7) was obtained. Through the tail vein, injection of the nanoparticulate contrast agent (ExiTron nano 12 000; 100 *μ*l; Miltenyi Biotech, Bergisch Gladbach, Germany) was performed to enhance imaging. With the animals in a supine position, light anesthesia was initiated (1.25% isoflurane). Subsequently, a micro-CT scanner (MicroSPECT4CT; MILabs, Houten, the Netherlands) using retrospective cardiac and respiratory gating was utilized. As presented in Bai et al (2024), we focused on the imaging space ranged from the renal vessels to below the bifurcation of the aorta/IVC, set a cone beam filtered back projection algorithm to 20 *μ*m effective voxel size, and performed Micro-CT with a 50 kVp Xray tube voltage, 430-lA tube current, 20 ms per frame, 360 angle, and 0.75 increments per view.

### 2.4. Heavy metal staining—sample preparation for high-resolution micro-CT imaging of tissue structures

Non-histology samples underwent modified heavy-metal based staining protocol originally utilized for electron microscopy (Hua et al 2015). The IVC-fistula-aorta complex was extracted from the mouse and underwent serial washes with 0.2 M cacodylate buffer. It was then subsequently immersed in 2% osmium tetroxide (OsO_4_) aqueous solution buffered with 0.2 M cacodylate (pH 7.4) at room temperature for 90 min. The staining buffer was replaced by 2.5% ferrocyanide in 0.1 M cacodylate buffer (pH 7.4), and then incubated at room temperature for 90 min. It was subsequently incubated in 4% pyrogallol (saturated aqueous solution at room temperature at 40 °C for 60 min, followed by 2% unbuffered OsO_4_ aqueous solution at room temperature for 90 min, and 1% uranyl acetate (UA) aqueous solution at 4 °C overnight. All samples were rinsed with filtered water between each step. Roughly 12 h later, samples were warmed up to 50 °C for 120 min. After being washed twice in filtered water at room temperature for 30 min, the tissue was incubated in a lead aspartate solution at 50 °C for 120 min. Lead aspartate solution was prepared by dissolving 0.066 g lead nitrate in 10 ml 0.03 M aspartic acid and pH adjusted to 5 with 1 N KOH. The tissue was then again washed with filtered water.

For embedding, samples were dehydrated through a graded ethanol series (25%, 50%, 75%, 100%, 30 min each, all cooled at 4 °C) into polyphenylene oxide (PPO; 3 × 100%, 30 min at room temperature), followed by epoxy-monomer infiltration by immersion into 1:1 mixtures of PPO and resin (Embed 812, DDSA, NMA) at room temperature for 12–24 h on a shaker. Samples were then incubated in 100% resin, then placed in embedding molds in an oven (60 °C) for 48–72 h. This method produces permanently stained and fixed samples that can undergo repeat cycles of high-level imaging.

### 2.5. High-resolution micro-CT imaging of tissue structures

Heavy metal stained samples (IVC-fistula-aorta complex) were imaged at the 2-BM-B bending magnet beamline at the Advanced Photon Source (Argonne National Laboratory) (Nikitin et al 2022). This parallel-beam micro-CT set-up integrated with an Optique Peter microscope system enables sub-micron imaging. X-rays with an energy of 30 keV were converted into visible light that were magnified using a 5x objective lens (0.70 *μ*m/pixel resolution) with 50 *μ*m scintillator with 25 *μ*m scintillator. 2120 projections were collected over 180° with 0.3 s exposure time. The projection size covered 3456 × 3452 pixels with a pixel size of 3.45 *μ*m. Raw data was saved to HDF5 files with EPICS *areaDetector* and reconstructed with TomocuPy and saved as a TIFF stack (Nikitin 2023).

### 2.6. Histology

Histology was obtained using the same methods previously described in Bai et al (2024). First, mice were euthanized. Second, through the left ventricle and under physiological pressure, perfusion with normal saline followed with 10% formalin was performed following with the extraction of the AVF, the embedding in paraffin, and the staining using Hematoxylin and eosin (H&E) and Elastin van Gieson (EVG). A part of the samples was paraffined in a longitudinal direction and cut in 5 *μ*m thickness while staining with H&E and EVG

### 2.7. Construction of three-dimensional AVF geometry

The computational model was defined using the vein-fistula-artery geometry extracted from the *in vivo* CTA scans and BCs from ultrasound measurements performed on day 7 after fistula creation. Raw Digital Imaging and Communications in Medicine (DICOM) images (figure 1(D)) were imported into Simpleware ScanIP (Synopsys, Exeter, United Kingdom) and the geometry was segmented by isolating the artery, vein, and fistula from the background noise (figure 1(E)).

The baseline fistula geometry was manually manipulated to create additional models of fistula stenosis and central venous stenosis. Fistula stenosis was created by decreasing the cross-sectional area of the fistula lumen within Simpleware. Central venous stenosis was modeled by decreasing the cross-sectional area of the vein lumen distal (cranial) to the fistula.

### 2.8. CFD model of AVF

The computational model was created by importing the final segmented geometries from Simpleware into Xflow (Simulia, Dassault Systems). Xflow is a particle-based Lattice Boltzmann kinetic solver that uses a proprietary particle-based Langrangian approach to solving CFD problems. Xflow uses wall-modeled large eddy simulation approach to turbulence modeling by providing a consistent local eddy-viscosity and near wall behavior (XFlow 2021x User Guide 2011).

We defined pressure and velocity inlet/outlet BCs for the artery and vein. The velocity BC for the artery (cranial) and vein (caudal) inlets were obtained from ultrasound mean arterial velocities and peak venous velocities measured *in vivo* (bolded values, table 1).

The time step was set to 2.5 * 10^−6^ s, simulation time 3 s. We utilized an adaptive mesh refinement algorithm with the finest mesh set to 3.25 * 10^−5^ m. We ran the model on The Center for Research Informatics at the University of Chicago Biological Sciences Division computing cluster. We utilized 30 CPUs. The domain was discretized into about 300 000 mesh elements. The simulation took about 72 h in real time.

**Figure 2.**
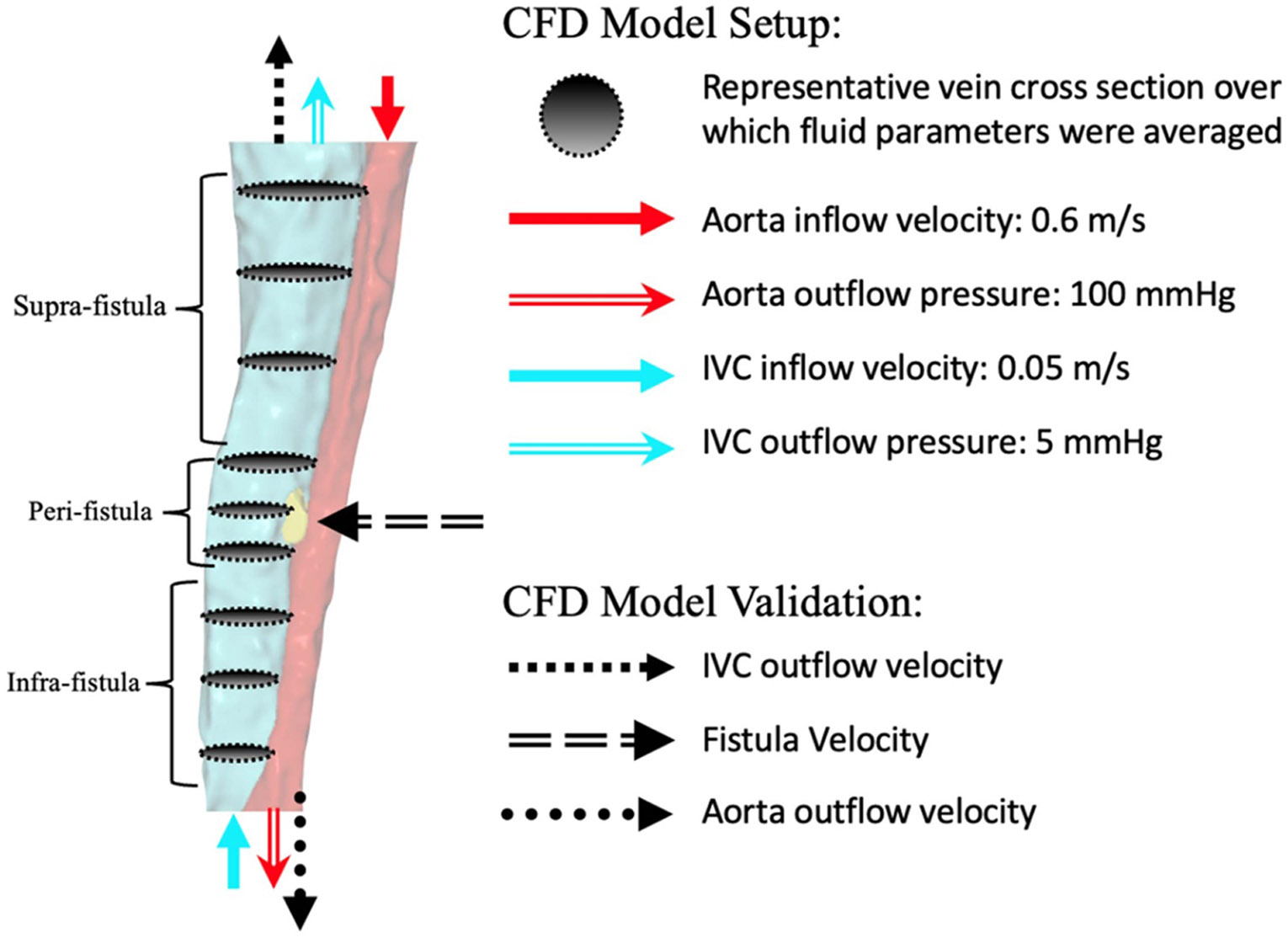
CFD model setup with velocity and pressure boundary conditions applied to the artery and vein inlets and outlets. Characterization of the flow field was averaged at varying cross sections along the vein (depicted by the grey cross-sectional oval).

There was no significant difference between day 7 and day 21 *in vivo* arterial and venous velocities. Of note, *in vivo* mean arterial velocity was used for the CFD arterial BC to account for the changes in velocity between systolic and diastolic waveforms. Hence, CFD arterial inlet BC was set at −0.8 m s^−1^ and the CFD venous inlet BC was set at 0.05 m s^−1^ (figure 2). The pressure BC for the artery (caudal) and vein (cranial) outlets were defined as follows: artery outflow pressure was assumed average physiologic blood pressure (100 mmHg) (Mattson 2001) and vein outflow pressure was assumed to be average central venous pressure (Xie et al 2014). Blood was modeled as a Newtonian fluid, with density 1060 kg m^−3^, temperature 288.15 K, viscosity 0.0035 Pa*s, thermal conductivity 0.0243 W*(m*K)^−1^, and specific heat capacity 1006.43 J*(Kg*k)^−1^.

We verified the computational stability and mesh independence of our simulation utilizing our non-stenotic AVF geometry (figures S1 and S2). Furthermore, to ensure that our conclusions regarding turbulence intensity were not unduly impacted by the assumption of a Newtonian fluid model we also ran the non-stenotic AVF model utilizing a Carreau–Yasuda viscosity model (figure S3). The Carreau–Yasuda model scales the fluid viscosity between limits of shear rates thereby allowing for distinct viscosities at low and high shear regions.

In the initial CFD simulations, arterial velocity was assumed to be constant. In later simulations, arterial velocity pulsatility was simulated using a 5-mode Fourier series (Prabu et al 2021).

(1) $$\nu (t)=a_{0}+\sum_{k=1}^{5}(a_{k}\;\cos(kwt)+b_{k}\;\sin\;(kwt))$$

Where

$$a_{0}=0.4248\;a_{1}=-0.06805,b_{1}=0.1644,\;a_{2}=-0.08076,b_{2}=-0.00885,\;a_{3}=-0.04299,b_{3}=-0.0357,\;a_{4}=0.01435,b_{4}=-0.02632,\;a_{5}=0.01557,b_{5}=-0.005171,\;\;w=14.77.$$

### 2.9. Characterization of the flow field

To characterize the model’s hemodynamics features, outputs from CFD simulations including velocity, static pressure, and turbulence were averaged along varying cross-sections along the length of the vein (figure 2).

The wall-adapting local eddy-viscosity (WALE) model is employed to calculate turbulence. This method was chosen to capture the possible turbulent and laminar flow regimes that may exist close to and far from the walls of the simulated system. WALE examines eddies near the boundaries in fluid flow simulations and adjusts to account for the turbulence adjacent to the fluid domain boundary. It incorporates the asymptotic nature of the turbulent boundary layer when the layer is solvable directly, and it does not introduce artificial turbulent viscosity in the shear regions away from the wake (Ducro et al 2007, Nicoud and Ducros 1999, Yin and Li 2015). WALE model for real flows in complex geometries is formulated using the system of equations:

(2) $$\nu_{t}={\left(c_{w}\Delta \right)}^{2}\frac{{\left({Ȿ}_{ij}^{d}{Ȿ}_{ij}^{d}\right)}^{3∕2}}{{\left({\bar{S}}_{ij}{\bar{S}}_{ij}\right)}^{5∕2}+{\left({Ȿ}_{ij}^{d}{Ȿ}_{ij}^{d}\right)}^{5∕4}}$$

where $\nu_{t}$ is the eddy-viscosity, $c_{w}$ is the WALE constant set to the default value of 0.2, Δ is the subgrid characteristic length scale, ${\bar{S}}_{ij}$ is the deformation tensor, and ${Ȿ}_{ij}^{d}$ is defined as follows:

(3) $${Ȿ}_{ij}^{d}=\frac{1}{2}\left({\bar{g}}_{ij}^{2}+{\bar{g}}_{ij}^{2}\right)-\frac{1}{3}\delta_{ij}{\bar{g}}_{kk}^{2}$$

(4) $${\bar{g}}_{ij}^{2}={\bar{g}}_{ik}{\bar{g}}_{kj}$$

where ${\bar{g}}_{ij}$ is the velocity gradient tensor, calculated by taking the gradient of the velocity vector, and $\delta_{ij}$ is the Kronecker symbol. Note that eddy viscosity ($\nu_{t}$) represents the effect of velocity fluctuations ($u^{\prime }$) on the stress in a fluid. $\nu_{t}$ is not a physical property of the fluid, and it depends on the intensity of the turbulent velocity fluctuations ($u^{\prime }$).

Turbulence intensity T (in %) is defined as the ratio between the root-mean-square of the turbulent velocity fluctuations ($u^{\prime }$) and the mean velocity (U) computed from the three mean velocity components ($U_{x}$, $U_{y}$, $U_{z}$):

(5) $$T=\frac{u^{\prime }}{U}$$

where $u^{\prime }$ and U are defined as:

(6) $$u^{\prime }=\sqrt{\frac{1}{3}\left({u^{\prime }}_{x}^{2}+{u^{\prime }}_{y}^{2}+{u^{\prime }}_{z}^{2}\right)}=\sqrt{\frac{2}{3}k}$$

(7) $$U=\sqrt{U_{x}^{2}+U_{y}^{2}+U_{z}^{2}}.$$

Note that k is the turbulent kinetic energy.

Unlike a physical property of the fluid, eddy-viscosity is the effective viscosity of turbulent flow, essentially quantifying the mixing effects caused by the velocity fluctuations ($u^{\prime }$). Turbulence intensity (T) is a measure of the velocity fluctuations ($u^{\prime }$) relative to the mean flow velocity (U).

### 2.10. Agent based modeling

To model NH as a function of turbulence, ABM was employed. ABM is a computational method used to simulate interactions of autonomous decision-making entities, called agents (Corti et al 2021). Each agent behaves based on a set of rules. The purpose is to assess the agents’ dynamic effects on a system as a whole as the simulation proceeds (as the results of the rule-based agent activities compound) (Bonabeau 2002).

The rules in this ABM were defined by the turbulence outputs from the CFD simulation. In the fluid simulation, the vein was equally divided into cross-sectional areas normal to the direction of fluid flow. Each cross-section was spatially defined by x, y, z coordinates. At each x−y−z position within individual cross-sections, characteristics of the flow field including velocity, static pressure, and turbulence were extracted. The ABM superimposed a 100 × 100 cell lattice onto each cross-section. Each lattice represented one venous cross-section, and each lattice cell was defined by the interpolated turbulence at each x−y−z coordinate within that cell area. Note that the number of lattice positions are the same across cross-section, thus the physical area that each grid cell represents scales with the venous radius at each cross section.

Each lattice position housed one ABM agent. Two types of agents were included in the ABM model: endothelial cell (EC) agents and vascular smooth muscle cell (VSMC) agents. Each agent’s behavior in its lattice position was based on three factors: location (space), age (time), and turbulence (hemodynamic effect). During initialization of the ABM, EC cells were generated on the boundary of the lattice with radius 20 (in units of grid cells), followed by 10 layers of VSMC cells. Turbulence was fed into the model from the CFD simulation (in units of % intensity—see equation (5)), interpolated to each lattice point in the interior of the circle of radius 20 using nearest neighbor method. Outside of the circle, the turbulence values were set to 0. The turbulence values remained the same throughout ABM simulation. This one-way coupling of ABM and CFD reduces the level of computational complexity while allowing the hypothesis that turbulence drives cellular responses leading to NH to be tested. After initialization, the model proceeded in steps (100 steps in total), with each step corresponding to an increase of 1 unit in age. Two probabilistic processes are considered in each step:

1. EC agent degradation: Each EC agent contains a probability of degradation of:
(8) $$P_{\text{EC}}(T)=\frac{1}{1+e^{-c_{\text{EC}}(T-T_{\max})}}$$
  T is the local turbulence at the position of the EC agent
  In each step, a random number p between [0, 1] is drawn for each EC agent and the EC age is determined. If the random number is lower than $P_{\text{EC}}$ and EC age is smaller than a critical value, EC degradation occurs and the EC cell is removed from the model. This process allows for VSMC differentiation taking over the degraded EC cell’s position, marking the start of the inflammation process.
2. VSMC agent differentiation: Each VSMC agent contains a probability of dividing of:
(9) $$P_{\text{VSMC}}(T)={\left(\frac{1}{1+e^{-c_{\text{VSMC}}(T-T_{\max})}}\right)}^{2}$$
  T is the local turbulence at the position of the VSMC agent.
  Using a similar algorithm for EC agent presented above, each VSMC agent reads the turbulence value at its cell position in the lattice, calculates the dividing probability, determines VSMC’s age, and selects a random number p between [0, 1]. To capture VSMC’s differentiation, additional calculations are performed for VSMC agents including updating the VSMC’s age for dividing and newly generated agents and determining the unoccupied spaces surrounding the VSMC agent. The VSMC agent can only divide if all of the three following conditions are satisfied:
  - The selected random number p is lower than the calculated $P_{\text{VSMC}}$.
  - The VSMC agent’s age does not exceed a critical age value. This is to ensure simulation convergence.
  - There is at least one unoccupied neighboring grid cell. If more unoccupied spaces are present, a random neighboring cell is chosen to house the new agent.

When division occurs, a new VSMC agent is placed on the chosen neighboring cell. Both the new and original VSMC agents’ ages are set to 0.

**Figure 3.**
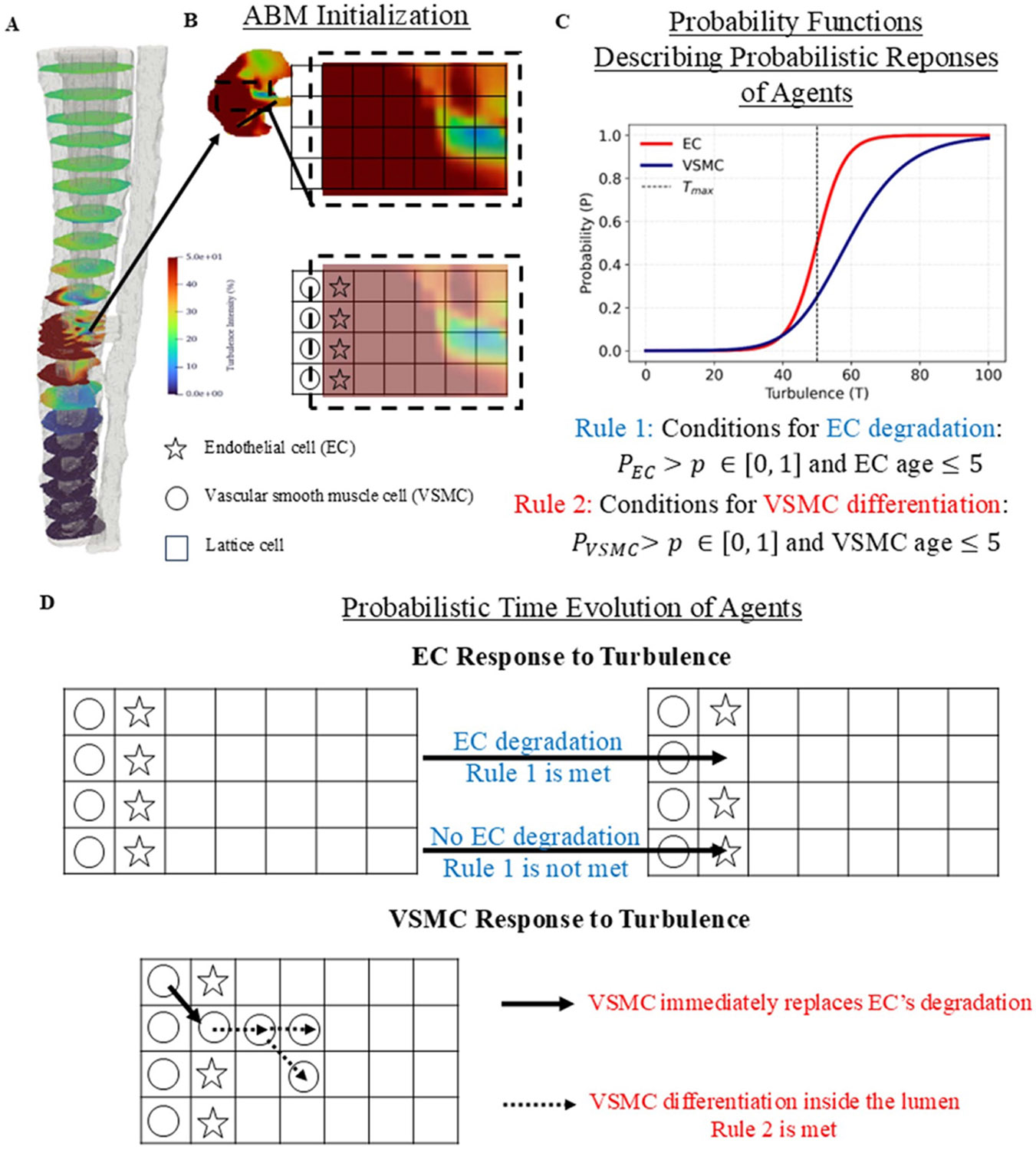
Example of agent-based model (ABM) of a cross section of vein at the peri-fistula region. First, the vein is divided into multiple cross sections normal to the direction of the flow velocity (A). Turbulence intensity within each slice is depicted by the color bar, with red and blue areas representing high versus low turbulence intensity respectively. For ABM’s initialization, a lattice is overlaid on the venous turbulence map, and the perimeter cell is pre-populated by layers of VSMC (circle) and endothelial cells (star) (B). To describe the responses of EC and VSMC to turbulence, two probabilistic rules are used to decide if EC degradation or VSMC differentiation occurs. Representative plots for EC degradation’s (red curve) and VSMC differentiation (blue curve) probability P as functions of local turbulence and the two rules using the optimal parameter set are shown in (C). The model evolves following the rules for EC degradation and VSMC differentiation (D).

The critical age value for both VSMC and EC agents were set to 5. Both probability functions for EC degradation and VSMC differentiation describe an S-shaped curve where the value of $P_{\text{EC}}$ or $P_{\text{VSMC}}$ transitions smoothly from 0 to 1 as turbulence (T) increases. The equations calculating $P_{\text{EC}}$ and $P_{\text{VSMC}}$ are sigmoid functions, which are used in similar biological context to describe the relation between mechanical stress and cell damage (Zahedmanesh et al 2014). $T_{\max}$ presents the midpoint or inflection point of the logistic curve. The square factor in the equation for $P_{\text{VSMC}}$ reflects the observation that to match *in vivo* response, the probability of differentiation of VSMC cells must not converge to 1 too quickly as turbulence increases. This is reflected in a more gradual slope in comparison to $P_{\text{EC}}$ pass the inflection point, as illustrated in figure 3(E).

The above interpretable two-rule ABM model is designed to test the hypothesis that turbulence driven EC death and VSMC differentiation can lead to the spatial distribution of NH observed in animal models. To achieve this the model was iteratively tuned by varying $T_{\max}\in \{50,55,65,80,100\}$. The optimal parameter set selected was $c_{\text{EC}}=0.25$, $c_{\text{VMSC}}=0.1$, and $T_{\max}=50$. An illustration of these rules is depicted in figure 3.

To quantify the amount of stenosis predicted at the level of the fistula using the ABM model, we calculated the percent of the venous lumen that was filled with VSMC cells after running the ABM model. This was compared to the area of intimal hyperplasia in the microscopy images, approximated using ImageJ software.

## 3. Results

### 3.1. Successful modeling of *in vivo* fistula and its perturbed hemodynamics

#### 3.1.1. Simulated velocity profiles and validation

The CFD model using the pressure and velocity BCs (see table 2) as described in the Method Section was first validated against ultrasound data (shown in table 1) in the mouse fistula system. As shown in table 2, the simulated velocities from the CFD model at corresponding ultrasound measured locations are in good agreement with experimental measurements. Specifically, when comparing tables 1 and 2, the simulated vein, artery, and fistula outlet velocities (0.115 ± 0.0506, −0.116 ± 0.0620, and 2.304 ± 0.687) are within one standard deviation of the measured *in vivo* velocities (0.27 ± 0.087, −0.31 ± 0.090 (peak velocity), 3.676 ± 1.958), respectively.

**Figure 4.**
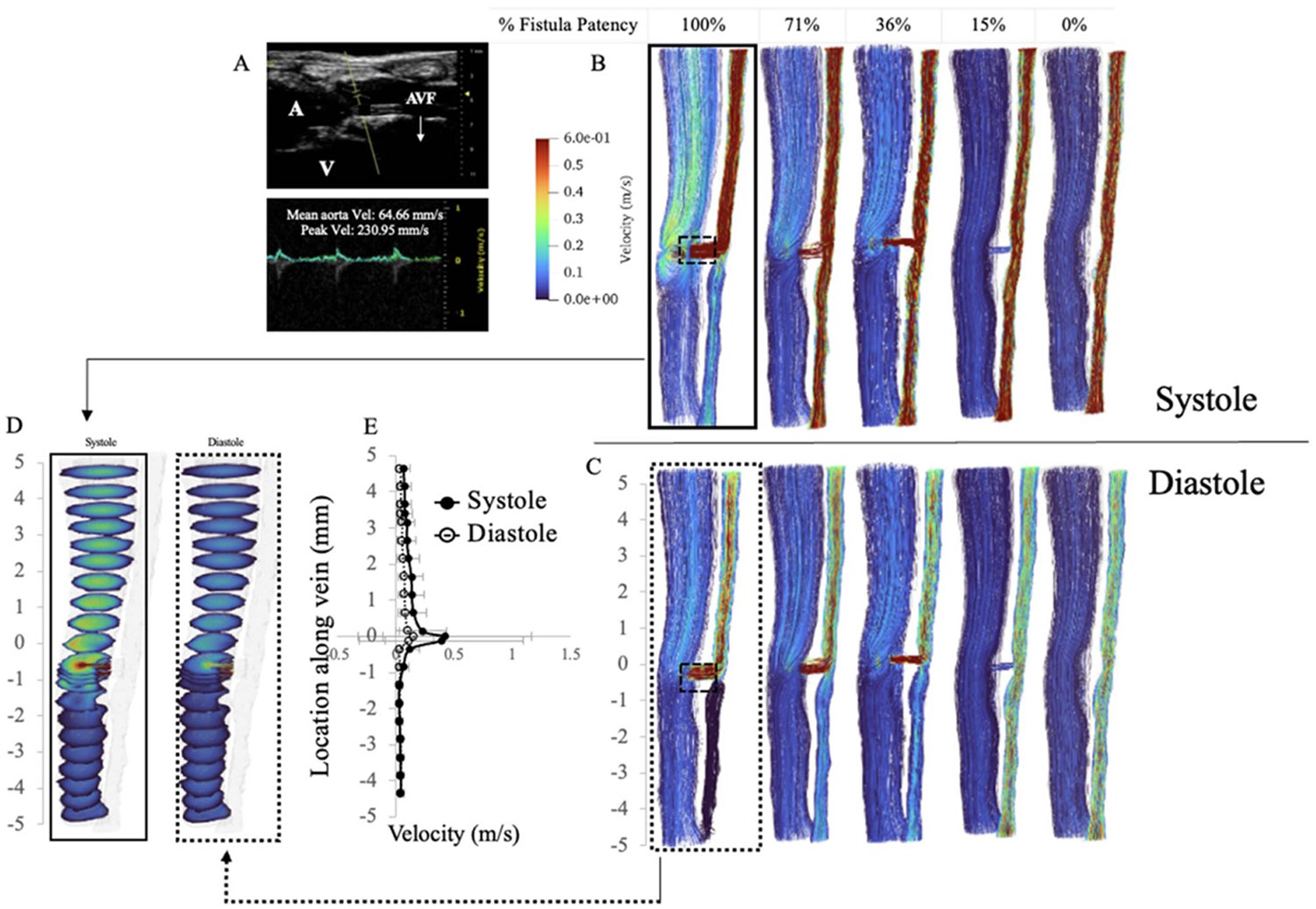
(A) Ultrasound measurement of velocity in the artery distal (caudal) to the fistula. (B), (C) Velocity streamlines in the vein-AVF-aorta complex as a function of fistula patency in the arterial systolic (B) and diastolic (C) cardiac phases. D) Cross sectional slices of the venous bulk fluid velocity when the fistula is 100% patent. (E) Velocity averages (and standard deviation) at each cross-sectional slice through the bulk fluid.

The velocity streamlines of the AVF model are shown in figure 4. Figures 4(B) and (C) compare the velocity streamlines as a function of fistula patency (100% patent, 71%, 36%, 15%, 0%) at systole and diastole, respectively. When the fistula is 100% patent (4(B) and (C), left), the bulk flow is diverted from the aorta and into the vein. With the fistula patent, velocity in the distal aorta dramatically decreases, as most of the flow is rerouted through the fistula and into the vein. This observation is confirmed with *in vivo* velocity ultrasound measurements in the distal aorta (figure 4(A), distal aortic velocity 64 mm s^−1^). As the fistula becomes more stenosed, the bulk flow is diverted from the stenosed fistula and back into the distal aorta, restoring native physiologic arterial/venous flow once the fistula is completely closed (figures 4(B) and (C), right-most streamlines). While we did not have direct comparison of *in vivo* velocities in stenotic AVFs to our CFD, the decreasing trend in in velocity with percent stenosis is consistent with clinical observations where fistula flow significantly decreases once >90% stenosis occurs. Velocity averaged at incremental venous cross sections (figure 4(D)) demonstrates a velocity peak at the level of the fistula, and sequential tapering of velocity further from the fistula (figure 4(E)). Additionally, when the inlet arterial velocity BC defines pulsatile flow (equation (1)), the venous peak velocity (y=0 in figure 4(D)) is greater in systole than when the artery is in diastole. Overall, the velocity results from our CFD simulations show strong agreement with experimentally observed values, validating that the correct hemodynamics are captured in this rigid-wall CFD simulation of an AVF. Finally, the results showed little variation with incorporation of the Carreau–Yasuda model as compared to the Newtonian fluid model (figure S3).

#### 3.1.2. Simulated static pressure and turbulence profiles and validation

**Figure 5.**
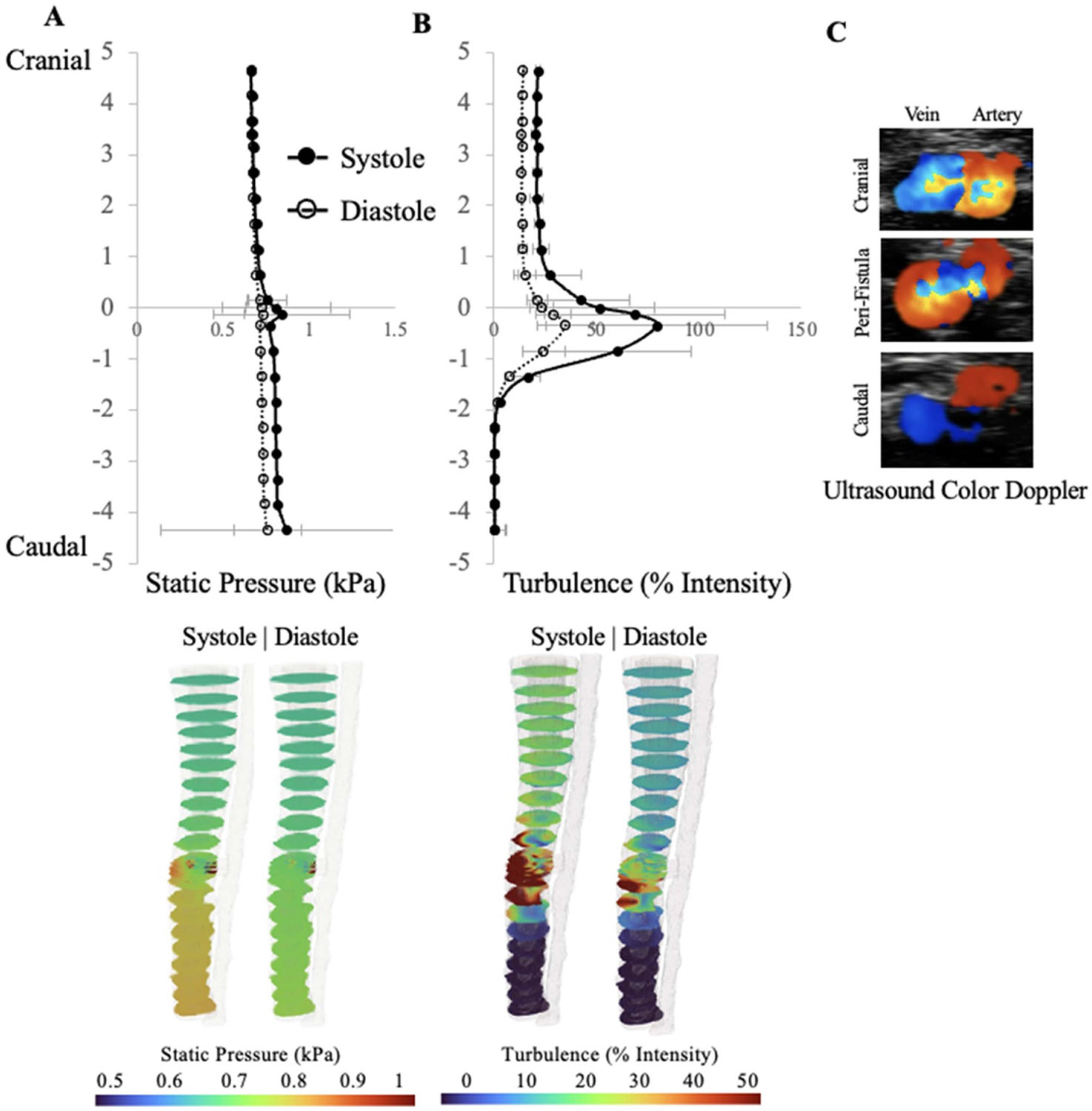
(A) Static pressure (*x* axis) along the length of the vein (*y* axis, with *y* = 0 at the perifistula region, positive y values are cranial to the fistula location and negative y values are caudal to the fistula location. (B) Turbulence intensity. (C) *In vivo* color Doppler images of flow in the vein and artery above and below the fistula, as well as in the peri-fistula region. Red/blue indicates flow towards/away from the ultrasound transducer, respectively.

While velocity is easily quantitated *in-vivo* with duplex measurements, spatial velocity fluctuations (turbulence) and spatial distributions of pressure are not. CFD however provides easy access to these fluid parameters. The static pressure and turbulence profiles were analyzed at incremental cross sections along the venous bulk fluid. The static pressure and turbulence averages were plotted along the length of the vein (figures 5(A) and (B)). Static pressure within the vein does not change in the presence of the fistula except for a very small increase in JAA pressure seen mostly at systole (5(A)). This is consistent with the known hemodynamics of an AVF where the venous system, functioning as a very large low-pressure reservoir, easily absorbs the excess volume coming through the fistula without much of a change in central venous pressures, i.e. the principle of compliance, which is the change in volume divided by the change in pressure.

While pressure changes weakly, turbulence intensity strongly varies along the vein with a peak at the JAA (y=0). When the arterial inlet BC is pulsatile flow, (equation (1)), turbulence is dramatically increased in the peri-fistula venous region when the artery is in systole compared to diastole (5(B)). Just distal to the fistula, there is some turbulence in the vein, but further distal to the fistula, turbulence is nearly negligible. Cranial to the fistula, the turbulence gradually dissipates over a length of ~1000 *μ*ms to reach a new low turbulence steady state. *In vivo* color Doppler ultrasound (5(C)) depicts some venous turbulent flow above (cranial) to the fistula, significant turbulent flow at the level of the fistula, and laminar flow below (caudal) the fistula, which validates the observed CFD turbulence outputs.

Using this validated framework to accurately access the hemodynamics of the AVF, we next study the perturbed hemodynamics induced by venous stenosis in the supra-fistula vein. These distal stenotic regions are meant to model regions such as the Cephalic arch clinically known to be prone to stenosis. The system was modeled using the pulsatile velocity (equation (1)) for the arterial inlet BC. Venous stenosis causes an increase in venous turbulence cranial to the stenotic region.

### 3.2. Agent based modeling correlates turbulence with NH

Areas of increased turbulence (figure 5(C)) correlate to increased NH seen in histology and micro-CT observations (figures 7(A) and (B) respectively). Thus, turbulence values from both the non-stenotic and the stenotic vein computational models were used as inputs in ABM models to predict areas of NH. Using the ABM model that integrates two probabilistic rules to describe the cellular responses and interactions of EC and VSMC (see Method section), a strong correlation between the proliferation of VSMCs and the locations of high turbulence index exists for both the non-stenotic and stenotic veins (figures 7(C) and (D) respectively). Moreover, the areas of increased VSMC proliferation in the ABM model (7(C)) qualitatively correspond to increased NH seen in histology and micro CT observations (7(A) and (B)). ABM simulations test the hypothesis that NH starts with degradation of endothelium cells secondary to highly perturbed fluid dynamics in turn signaling VSMC cells to proliferate leading to areas of NH. *In vivo* models of NH (microscopy and histology results) strongly correlate with predictive models of venous thickening that are based on simulated fluid turbulence (figure 7(C) as compared to figures 7(A) and (B)). Additionally, prior evidence from the Dardik lab suggests that NH occurs at the level of the stenotic vein, which is also captured in our model (figure 7(d)).

At the level of the fistula, the ABM predicted 50%–80% of the venous lumen to be filled with VSMC cells. We compared this calculation with the actual percent of intimal hyperplasia in the vein *in vivo.* The ABM predicted extent of VSMC accumulation closely matched the degree of intimal hyperplasia *in vivo*. (figure S4)

## 4. Discussion/conclusion

NH is a potential major cause of AVF failure. The connection of a vein to the arterial systems exposes the thin-walled, compliant vein to the pressure and pulsatile velocity profile of the arterial system that is arguably much higher than what the vein is prepared to accommodate. The mechanical mismatch is a setup for disturbed flow within the vein that is postulated to correlate with focal intimal hyperplasia and subsequent failure of the fistula (Liu 1998, Chiu and Chien 2011). In this study, we developed computational models with CFD and ABM validated against experimental and imaging data to elucidate this postulated link between perturbed flow in AVF with and without stenotic veins with the development of NH. First, an animal-specific image-based CFD model for AVF is developed and compared against ultrasound measurements. This validated computational model allows the efficient quantifications of key hemodynamic parameters that are difficult to measure directly in small animal models, such as the velocity, pressure, and turbulence profiles along the venous segment of the AVF. Second, we coupled CFD turbulence outputs to an ABM model where two probabilistic processes were developed to describe endothelial degradation and VSMC proliferation. This one-way coupling CFD-ABM framework was used to test the hypothesis that endothelial death/VSMC proliferation driven by turbulence in AVF is associated with NH observed histologically.

**Figure 6.**
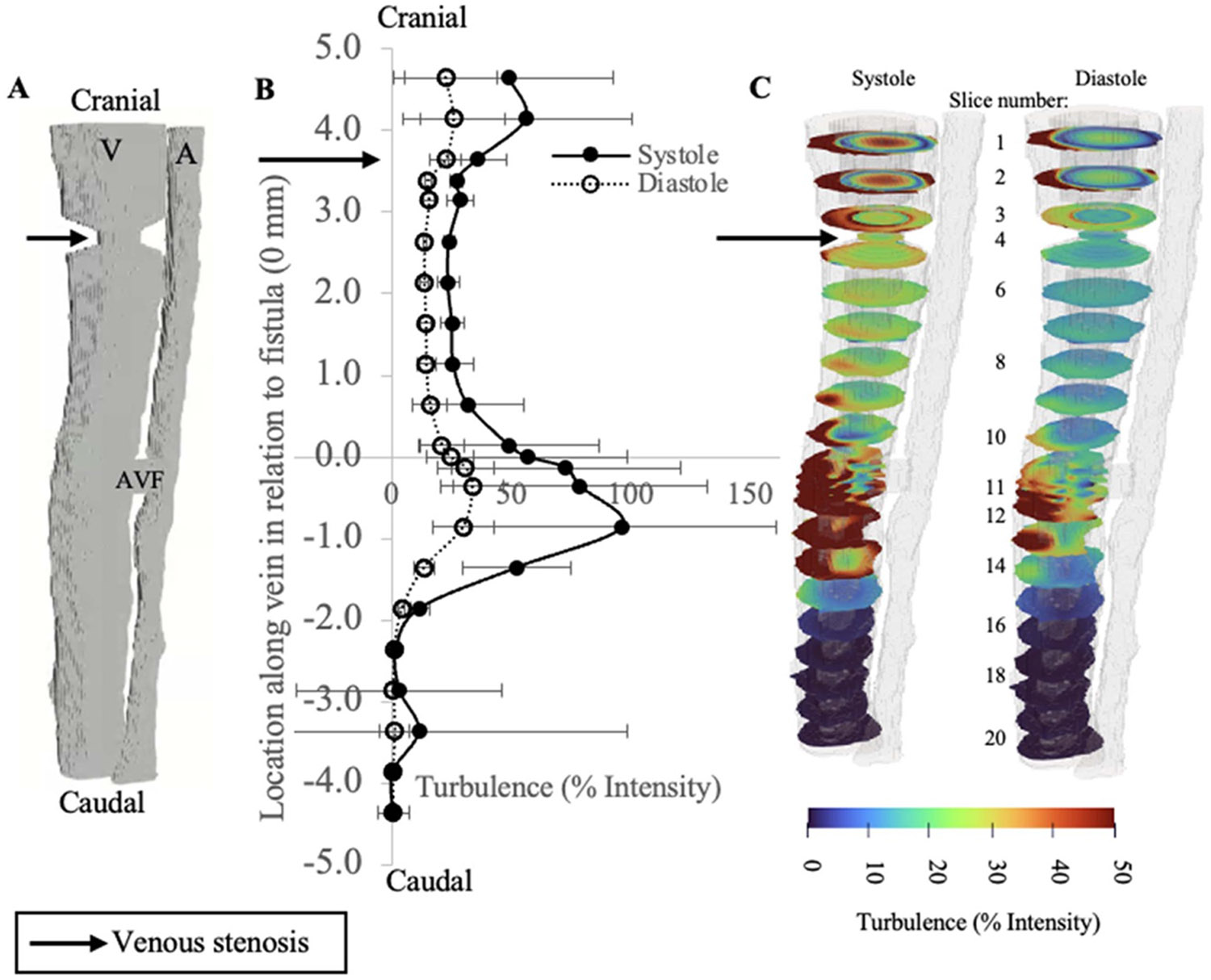
(A) 3D model of central stenosis in the supra-fistula vein (black arrow points to venous stenosis). (B) Quantification of turbulence (*x* axis) along the length of the vein (*y* axis, *y* = 0 at the level of the fistula). (C) Turbulence profiles at incremental cross sections labeled by slice number along the vein.

Our findings indicate that the JAA is particularly susceptible to non-homeostatic hemodynamic conditions, specifically elevated velocity and turbulence compared to other regions of the AVF (figures 4-6). Our CFD model shows a peak in velocity at the level of the fistula, consistent with prior CFD studies using murine models and micro-CT based CFD that showed a near doubling of blood flow through the AVF (Li et al 2025a). Additionally, these studies also showed the development of disturbed flow at the anastomosis which is consistent with our results that indicate high levels of turbulence at the JAA region (Li et al 2025a, 2025c). Other studies have shown that flow disturbance also increases at the juxta-anastomotic stenosis, which parallels our stenotic vein model prediction (Yang et al 2020). Blood flow disturbance is a predictor for vascular stenosis, and much research has been done to optimize the anastomotic angles of surgically created AVFs to maximize geometric parameters that may affect the AVF maturation process (Li et al 2025b).

**Figure 7.**
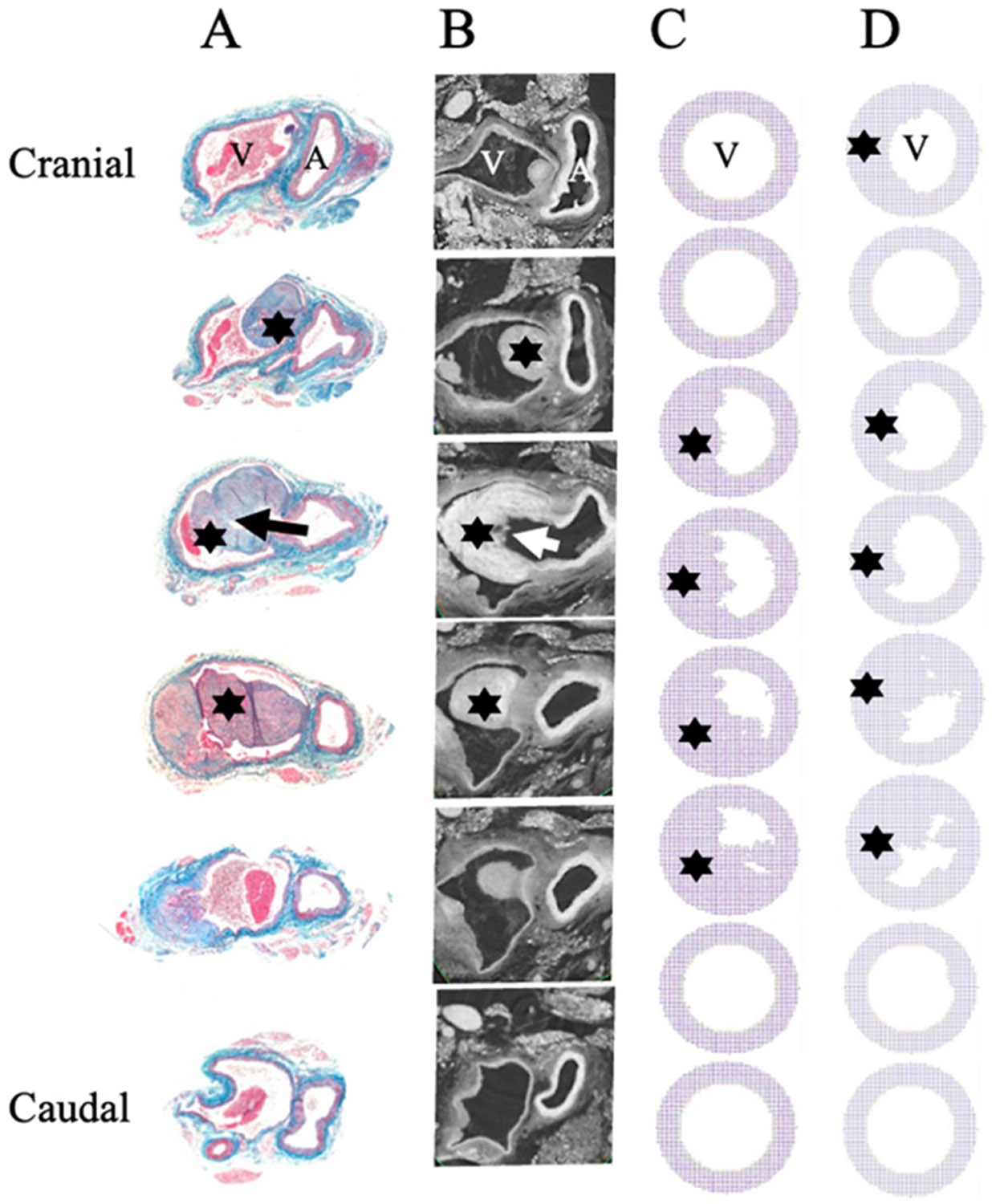
(A) AVF sections stained with Verhoeff–Van Gieson (EVG) from the aorto-IVC-fistula complex in the cranial to caudal direction at day 21; V: inverior vena cava; A: aorta. Electron microscopy (B) of AVF geometry correlates with histology-stained sections (A). Black/white arrow outline the fistula channel, black star notes areas of venous NH. Agent based modeling of predicted neointimal hyperplasia along the length of the vein when the fistula is patent (C) and when the vein is stenotic cranial to the fistula (D). Representative slices correlate to slices in the CFD model.

These disturbed flow conditions parallel *in vivo* development of NH particularly along a spatial gradient within the vein. The CFD model’s ability to replicate *in vivo* conditions strengthens the argument that these hemodynamic disturbances are not merely incidental but are likely driving pathological changes within the AVF that contribute to AVF failure. To test the hypothesis that turbulence contributes causally to NH, we implemented a simplified ABM using two probabilistic rules derived from turbulence intensity: one for EC degradation and one for VSMC proliferation. While the ABM is intentionally minimal and does not attempt to replicate all aspects of vascular remodeling, it provides a conceptual framework for exploring how localized turbulent stress might trigger cellular behaviors associated with NH. Notably, the spatial patterns of predicted NH in the ABM correspond qualitatively to regions of histological thickening (figure 7), indicating that the developed turbulence-driven rule set in this work can recapitulate key features of the observed biology. These findings support the idea that turbulence may play a direct and spatially specific role in initiating cellular responses that lead to AVF failure.

This model is limited by the use of CFD alone, without incorporating fluid-structure-interaction, thereby neglecting potential venous deformation and flow-structure coupling effects. However, several AVF studies also solely focus on CFD alone to simplify the problem and understand the fluid flow throughout the system (Ene-Iordache et al 2001, Kharboutly et al 2007, 2010, Sigovan et al 2013, Jia et al 2015, Prouse et al 2020, Northrup et al 2022). The model we describe in this paper serves as a basis for future work that will focus on how the flow field affects the venous deformation overtime and augments the turbulence, shear stress, and velocity profiles.

The results of this study provide insights into the hemodynamic factors that contribute to AVF failure, specifically highlighting the critical role of increased velocity and turbulence at the JAA. The observed correlation between elevated static pressure and the presence of venous stenosis underscores the importance of monitoring and managing hemodynamic conditions to mitigate the risk of AVF failure. Our study demonstrates that venous stenosis exacerbates turbulent flow, further contributing to the pathological environment that fosters NH. We see an increase in NH in the vein upstream of the fistula which is consistent with the *in vivo* observation in Dardik’s lab (Taniguchi et al 2020). This insight is crucial for developing targeted therapeutic strategies aimed at maintaining AVF patency.

This approach allows for the exploration of the complex biological responses to hemodynamic stress in a controlled, quantitative manner, providing a more comprehensive understanding of AVF pathology. A limitation of our study is the assumption of Newtonian flow in our CFD model. Blood is a non-Newtonian fluid, and this assumption may simplify the complex properties of blood, particularly in regions of disturbed flow where shear rates are low. Future studies should consider incorporating non-Newtonian flow characteristics to improve the accuracy of the hemodynamic simulations and provide a more nuanced understanding of the flow conditions within the AVF.

In conclusion, this work advances our understanding of the hemodynamic factors that contribute to AVF failure. By leveraging CFD and ABM, we have provided a detailed characterization of the disturbed flow conditions that may precipitate pathological changes within the AVF. These findings emphasize the need for ongoing research to explore potential interventions that can modify the hemodynamic environment of the AVF, thereby improving its longevity and reducing the incidence of failure. Future studies should aim to refine these models further and test potential therapeutic approaches in both preclinical and clinical settings, with the ultimate goal of enhancing the durability and function of AVFs in patients requiring chronic hemodialysis.

## Supplementary Material

supplementary data a

Supplementary material for this article is available online

## Figures and Tables

**Table 1.** *In Vivo* Ultrasound measurements at Day 7 and Day 21. The CFD velocity BC for the artery (cranial) and vein (caudal) inlets were obtained from ultrasound mean arterial velocities and peak venous velocities measured *in vivo* (bold).

Location	Day 7		Day 21	
	Mean velocity (m s^−1^)	Peak velocity (m s^−1^)	Mean velocity (m s^−1^)	Peak velocity (m s^−1^)
Artery, supra fistula	**−0.82 ± 0.37 (n = 8)**	−1.29 ± 0.589 (*n* = 11)	**−0.79 ± 0.12 (n = 10)**	−1.1 ± 0.37 (*n* = 13)
Artery, infra fistula (outlet)	Not recorded	−0.20 ± 0.0058 (*n* = 1^a^)	Not recorded	−0.31 ± 0.090 (*n* = 3)
Vein, supra fistula (outlet)	0.29 ± 0.14 (*n* = 8)	0.34 ± 0.16 (*n* = 11)	0.27 ± 0.087 (*n* = 10)	0.30 ± 0.16 (*n* = 13)
Vein, infra fistula	Not recorded	**0.066 ± 0.0055 (n = 1**^a^)	Not recorded	**0.054 ± 0.0066 (n = 2)**
Fistula	Not recorded	0.480 ± 0.00366 (*n* = 1)	3.676 ± 1.958 (*n* = 2)	2.994 ± 1.365 (*n* = 2)
Sham Artery	−0.169 ± 0.0413 (*n* = 4)	−0.461 ± 0.113 (*n* = 9)	−0.115 ± 0.035 (*n* = 6)	−0.324 ± 0.101 (*n* = 11)
Sham Vein	0.0632 ± 0.0342 (*n* = 4)	0.0773 ± 0.0475 (*n* = 9)	0.0441 ± 0.0186 (*n* = 6)	0.0651 ± 0.030 (*n* = 11)

a 1 mouse but 3 measurements.

**Table 2.** Boundary conditions used in the simulation model and post-simulation velocity measurements.

	Defined boundary condition (m s^−1^)	Simulated velocity from Computational model (m s^−1^)
Aorta, supra fistula	−0.8 (inlet)	−0.786 ± 0.380
Aorta, infra fistula (outlet)		−0.116 ± 0.0620
IVC, supra fistula (outlet)		0.115 ± 0.0506
IVC, infra fistula	0.05 (inlet)	0.040 ± 0.0169
Fistula		2.304 ± 0.687
Sham aorta		−0.841 ± 0.287
Sham IVC		0.0322 ± 0.00867

## Data Availability

All data that support the findings of this study are included within the article (and any supplementary information files).
