# Supplementary material for: Turbulence drives arteriovenous remodeling: an experimentally validated multi-scale model of neointimal hyperplasia
Source: Phys Med Biol. Author manuscript; Available in PMC 2026 Feb 18. (PMC12915627; doi:10.1088/1361-6560/ae1ac8)
Supplement: supplementary data a [file NIHMS2139597-supplement-supplementary_data_a.pdf]

Supplemental Material

Mesh Sensitivity Analysis:

To justify the resolution of our CFD model, we analyzed simulation stability and performed a mesh sensitivity analysis. First, the stability parameter calculated in XFlow allows the user to check for solution convergence. The stability parameter must satisfy the Courant-Friedrichs-Lewy condition, a fundamental requirement used in CFD time-marching schemes to ensure that the selected time step is appropriate for the physical system. The stability parameter must converge to ensure a stable system. (*XFlow 2021x User Guide*, 2011)

In Xflow, the stability parameter is a function of compressibility and numerical viscosity and is formulated by the following equations:

$$S = \frac{\max\,(S_{\upsilon}, S_{\nu}, S_{\rho})}{0.7} \qquad \text{(S1)}$$

where

$$S, Stability\ parameter$$
$$\nu, Kinematic\ viscosity$$
$$C_s = \frac{dx}{\sqrt{3}dt}, Numerical\ speed\ of\ sound$$
$$S_\nu = \frac{\nu}{C_s dx}, Numerical\ kinematic\ viscosity$$

$$S_\rho = \frac{2(\rho_{max} - \rho_{min})}{(\rho_{max} + \rho_{\min})}$$

To verify the stability of the simulation, we plotted the stability parameter over the duration of the original AVF model simulation (**Fig. S1A**). A stability parameter less than 1 indicates a stable simulation for the duration of the simulation. In addition, we plotted the mass flux through the system to additionally confirm that the model is at steady state (**Fig. S1B**). The outlet and inlet mass flux match each other, and the data is extracted from the final cardiac cycle when the simulation is at a steady state.

To ensure our simulation results were accurate and computationally efficient, we performed a mesh sensitivity analysis (see **Fig. S2**). We chose 4 different mesh densities to sample. We report the calculated slice average velocity magnitude along the AVF model for each mesh density. The error bars in the figure indicate the standard deviation of the data. The chosen mesh density for the paper is reported as medium in the figure. In the portion of the IVC labelled slices 1-10, the varying mesh density has little impact with the error bars overlapping. However, when we transition to the region of the fistula, we note that the low mesh density has a considerably lower velocity magnitude compared to the other mesh densities. In contrast, the three higher mesh densities all show overlapping error bars in the peri-fistula region. We chose the medium mesh density because of its considerable computational advantage compared to the medium-high and high mesh densities in terms of computational time (which is shown in hours in the figure). It is notable that even an increase in roughly 100,000 elements in between the medium and medium-high mesh

densities adds nearly 24 hours worth of computing time. The medium mesh density is able to capture the increased velocity at the peri-fistular region without adding exorbitant computational burden. As such, we chose to proceed with that for the remainder of our simulations.

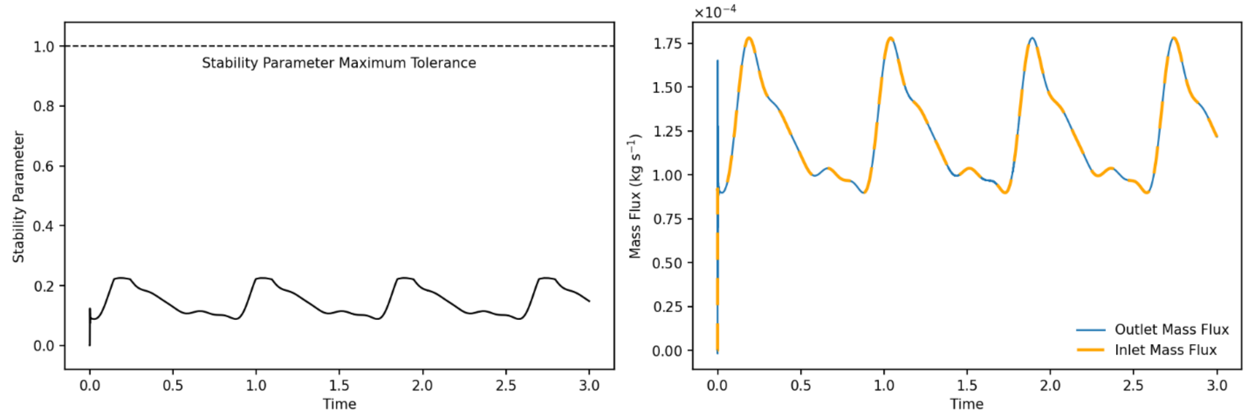

Fig S1: A) Stability parameter graphed as a function of simulation time. When the stability parameter is greater than 1.0, the stability of the simulation is compromised. B) The mass flux at the inlet equals the mass flux of the outlet, ensuring the system is in steady state.

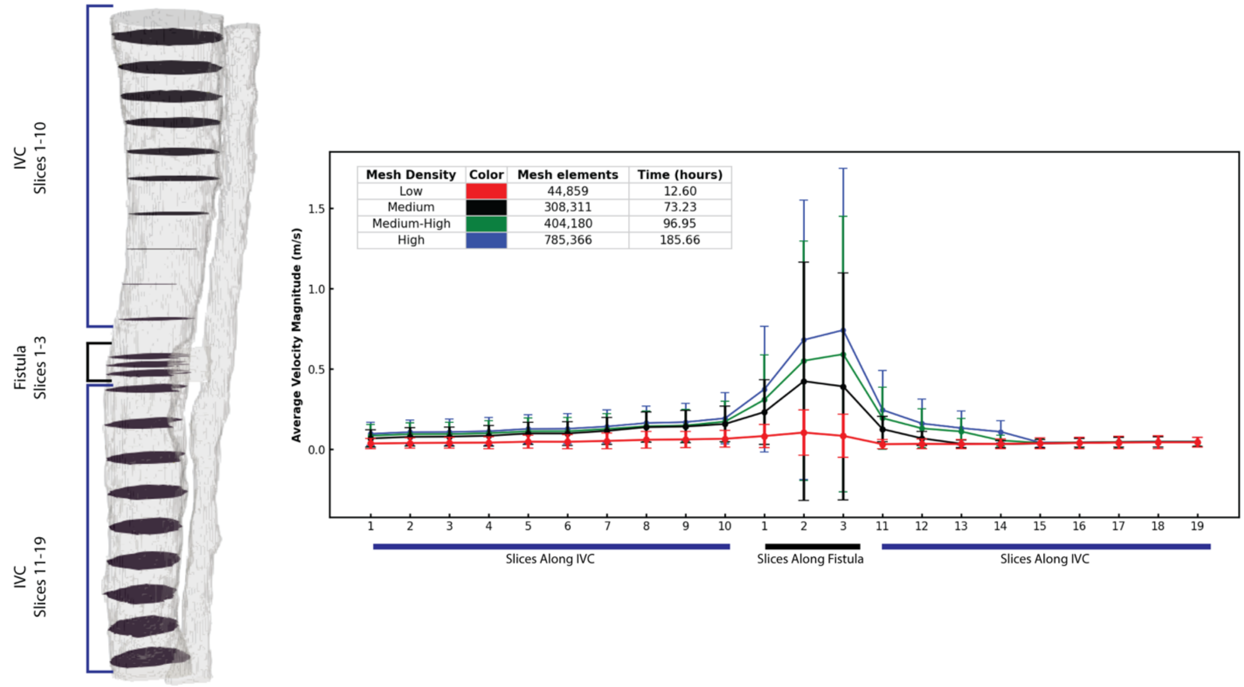

Fig S2: Mesh sensitivity analysis of the AVF simulation. Results from the original mesh (black) were compared with two finer meshes and one coarser mesh to evaluate the effect of mesh density on solution accuracy and computational cost. The selected mesh (black) demonstrated convergence without loss of accuracy.

Newtonian Assumption:

In this study, blood was modeled as a Newtonian fluid with constant dynamic viscosity of 0.0035 Pa *s. This assumption is widely used in simulations of blood vessels where shear rates exceed 100 s^{-1} and the non-Newtonian effects of red blood cell aggregation are negligible, such as in

arteriovenous fistulas.(Secomb & Pries, 2013; Zhou et al., 2023) While this simplification is reasonable in systems where shear rates are high, it may underestimate effects in regions of low shear, such as in large vessels and near vessel walls where blood behaves more like a non-Newtonian fluid. To address this limitation, we applied the Carreau-Yasuda model to capture non-Newtonian effects in the arteriovenous fistula system. We used our baseline simulation for comparison. We modelled the Carreau-Yasuda model as consisting of (Bernabeu et al., 2013; Liu et al., 2021; Lynch et al., 2022):

$$\mu(\dot{\gamma}) = \mu_\infty + (\mu_0 - \mu_\infty)(1 + (\lambda\dot{\gamma})^a)^{\frac{n-1}{a}}$$

Where:

$$\mu_\infty = 0.0035\, Pa * s$$
$$\mu_0 = 0.16\, Pa * s$$
$$\lambda = 8.2\, s$$
$$a = 0.64$$
$$n = 0.2128$$

Our results from this are shown in the figure below. Given the relatively high global shear rates in our system the Carreau-Yasuda model has little impact on both velocity magnitude as well as turbulence in most of our system. The portion of our IVC after the fistula (slices 11, 12, and 13) does show a slight discrepancy in the developed velocity magnitude which is expected given this is a low shear region in our system. Overall, there is not a significant impact in our data from adding in the Carreau-Yasuda model as compared to modeling blood as a Newtonian fluid.

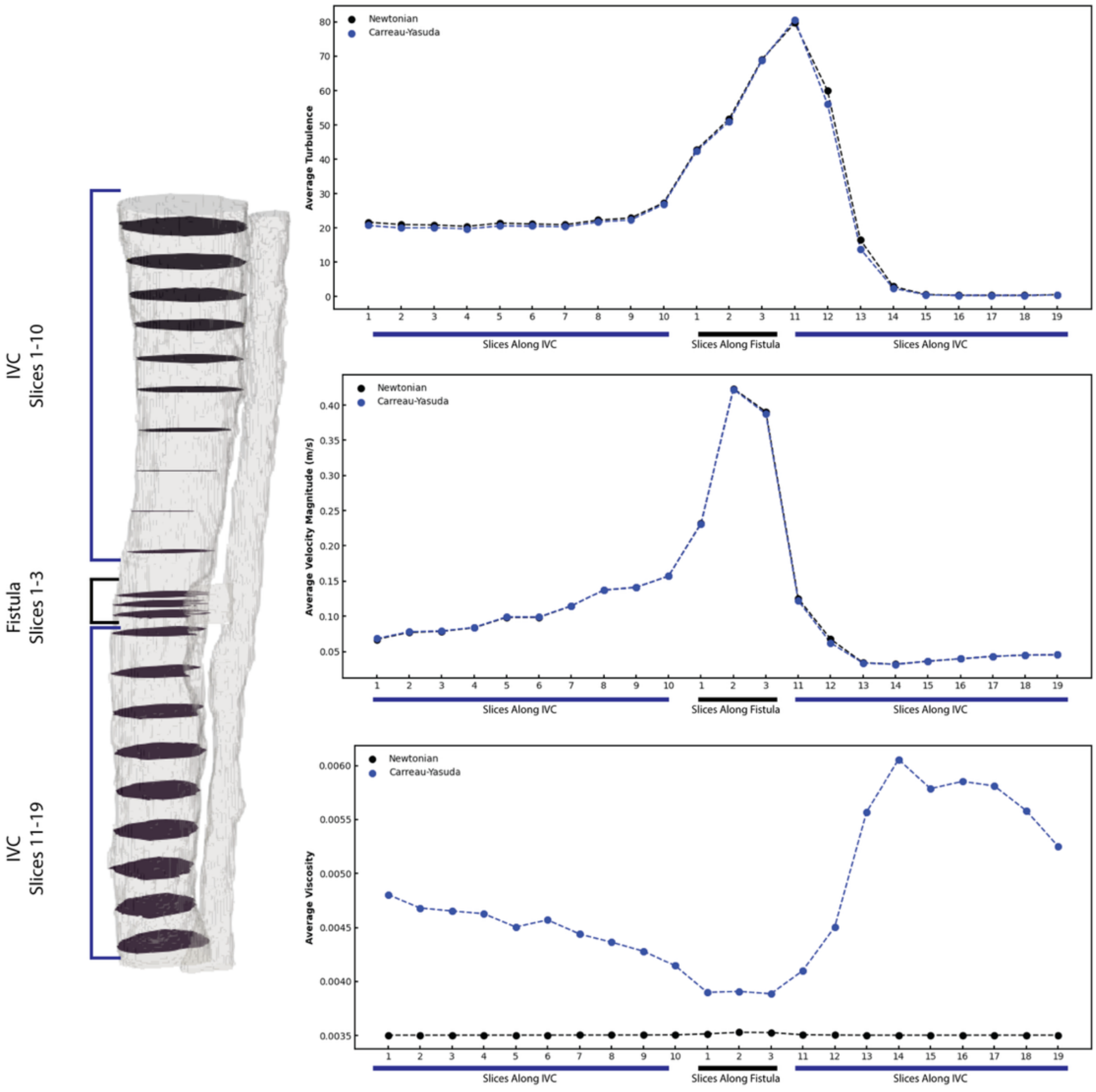

Fig S3: Comparison of Newtonian and Carreau-Yasuda non-Newtonian models in the AVF simulation. The velocity magnitude and turbulence distributions show minimal differences between the two models. Slight discrepancies are observed in the post-fistula IVC region (slice 11-13) corresponding to low-shear conditions. Overall, incorporation of the Carreau-Yasuda model underscored the appropriate assumption of Newtonian fluid.

<u>ABM VSCM Quantification:</u>

To evaluate the accuracy of the ABM prediction, we compared model-derived venous lumen stenosis (VSMC accumulation) with experimental measurements (from microscopy slices using ImageJ).

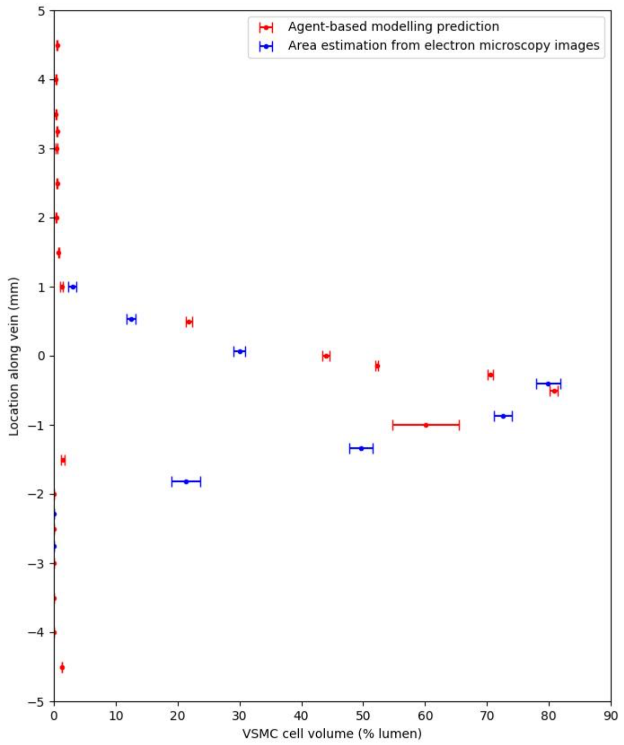

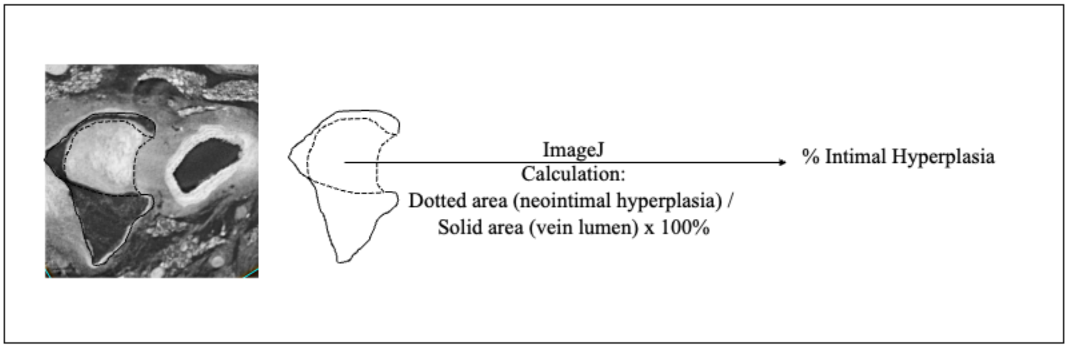

Fig S4: A) Comparing the percent of venous intimal hyperplasia (VSMC accumulation) between the ABM predictive model and *in vivo* neointimal hyperplasia quantified from microscopy slices using ImageJ. The red error bars represent the discrepancy between the Newtonian and the non-Newtonian simulations. Specifically, each red error bar corresponds to the difference in the predicted volume of VSMC accumulation in the venous lumen when the C-Y model was used in place of the Newtonian assumption. The red error bars illustrate the range within which our predictions may vary depending on whether shear-thinning effects are considered. The blue error bars represent human error when calculating VSMC infiltration using ImageJ. Overall, the ABM predictions closely reflected the experimental data, demonstrating the model's ability to predict neointimal hyperplasia. B) Schematic of how venous neointimal hyperplasia was quantified from microscopy slices using ImageJ software.

XFlow 2021x User Guide. 2011;

Zhou G, Chen Y, Chien C, Revaea L, Ferdous J, Chen M, et al. Deep learning analysis of blood flow sounds to detect arteriovenous fistula stenosis. Npj Digit Med. 2023 Sep 1;6(1):163.

Secomb TW, Pries AR. Blood viscosity in microvessels: experiment and theory. Comptes Rendus Phys. 2013 Jun;14(6):470–8.

Liu H, Lan L, Abrigo J, Ip HL, Soo Y, Zheng D, et al. Comparison of Newtonian and Nonnewtonian Fluid Models in Blood Flow Simula9on in Pa9ents With Intracranial Arterial Stenosis. Front Physiol. 2021;12:718540.

Lynch S, Nama N, Figueroa CA. Effects of non-Newtonian viscosity on arterial and venous flow and transport. Sci Rep. 2022 Nov 29;12(1):20568.

Bernabeu MO, Nash RW, Groen D, Carver HB, Hetherington J, Krüger T, et al. Impact of blood rheology on wall shear stress in a model of the middle cerebral artery. Interface Focus. 2013 Apr 6;3(2):20120094.
